# Supplementary material for: Content-rich biological network constructed by mining PubMed abstracts
Source: BMC Bioinformatics. 2004 Oct 8;5:147. doi: 10.1186/1471-2105-5-147 (PMC528731; doi:10.1186/1471-2105-5-147)
Supplement: Additional File 2 — The original results of the above study (non-essential files are deleted to keep the file size under the limit set by BMC bioinformatics). [file 1471-2105-5-147-S2.bz2 › chilibotAdditionalFile2/dip20/153ID1423597E548/html/CDK4_CCND1.html]

 


 **CDK4** and **CCND1** 
  
Found 880 abstracts in PubMed, retrieved 20.  
 

 What does Google say? 
 PDF only 
| .edu only 

---

**Interactive relationship** (e.g. stimulation, inhibition, etc)

**Inhibitory relationship**- Cytoplasmic cyclin D  [ **CCND1** ]  1 assembled with cyclin dependent kinase 4  [ **CDK4** ]  CDK 4  [ **CDK4** ] , and the CDK inhibitors p21Cip1 and p27Kip1.  Ref: 12872751 Kokubyo Gakkai Zasshi, 2003
- Rapamycin and its analogues also prevent cyclin dependent kinase CDK activation, inhibit retinoblastoma protein phosphorylation, and accelerate the turnover of cyclin D1  [ **CCND1** ] , leading to a deficiency of active  **CDK4**  cyclin D1  [ **CCND1** ]  complexes, all of which potentially contribute to the prominent inhibitory effects of rapamycin at the G1 S boundary of the cell cycle.  Ref: 12864941 Clin Breast Cancer, 2003
**Stimulatory relationship**- Increased expression, or stability, of cyclin D1  [ **CCND1** ]  molecules may cause sufficient  **cdk4**  activation to produce retinoblastoma protein phosphorylation independently of mitogenic signals.  Ref: 12834607 J Comp Pathol, 2003
**Neutral relationship**- The three human D type cyclins, cyclin D1  [ **CCND1** ] , D2 and D3 share the ability to bind to and activate  **cdk4**  and 6.  Ref: 12853979 Oncogene, 2003

**Non-interactive relationship** (e.g. studied together, co-existance, homology, etc.)

- On the other hand, EPA inhibited cyclin dependent kinase 4  [ **CDK4** ]   **CDK4**  activation and cyclin D1  [ **CCND1** ]  protein induction, a critical step for G1 S progression.  Ref: 12811822 J Cell Physiol, 2003
- These effects were highly specific, as the kinetics of cyclin D  [ **CCND1** ]   **cdk4**  activation was unaltered.  Ref: 12842876 J Biol Chem< MedlineTA>J Biol Chem, 2003
- Moreover, Rb phosphorylation, which lies immediately downstream of the cyclin D1  [ **CCND1** ]   **Cdk4**  p21WAF1 Cip1 complex, was blocked either by Sp1 overexpression or antisense p21WAF1 Cip1.  Ref: 12796485 J Biol Chem< MedlineTA>J Biol Chem, 2003
- In a series of 23 surgical biopsies of grade II and III oligodendroglioma, cyclin D1  [ **CCND1** ] , E, A, B1,  **CDK4**  6, CDK2, Cdc2 and p27 Kip.1 have been studied by immunohistochemistry and Western blot.  Ref: 12873740 Neurosci Lett, 2003
- The restored ZD AZ forestomach epithelium displayed strong expression of Bax, a proapoptotic protein, and weak staining of cyclin D1  [ **CCND1** ]  and its catalytic partner  **Cdk4** , key regulatory proteins controlling G 1 to S progression.  Ref: 12873989 Cancer Res, 2003
- Sp1 blocked assembly of cyclin D1  [ **CCND1** ]   **Cdk4**  p21WAF1 Cip1 complex formation whose integrity is critical for G1 > ; S transition.  Ref: 12796485 J Biol Chem< MedlineTA>J Biol Chem, 2003
- Arsenic increased expression of the P21 protein and decreased levels of cyclin A, cyclin B1 and cyclin D1  [ **CCND1** ] , but expression of CDK2,  **CDK4** , CDK6, and cyclin E were not affected.  Ref: 12883267 Pancreas, 2003
- Binding of these CKI s to both the cyclin D1  [ **CCND1** ]  CDK 4  [ **CDK4** ]  complex and the cyclin E CDK 2 complex were increased and the kinase activities of these complexes were reduced with increasing dietary corticosterone.  Ref: 12807724 Carcinogenesis< MedlineTA>Carcinogenesis, 2003
- No changes were observed in the expression levels of the G1 regulatory molecules cyclin D1  [ **CCND1** ]  and  **cdk4** , but cell cycle arrest and apoptosis induction was accompanied by accumulation of the cdk inhibitor p21 in both cell lines.  Ref: 12846895 Br J Haematol, 2003
- Levels of cyclin D1  [ **CCND1** ] , cyclin E, CDK 2 and CDK 4  [ **CDK4** ]  were reduced in carcinomas from corticosterone treated rats.  Ref: 12807724 Carcinogenesis< MedlineTA>Carcinogenesis, 2003
- Sp1 Inhibits proliferation and induces apoptosis in vascular smooth muscle cells by repressing p21WAF1 Cip1 transcription and cyclin D1  [ **CCND1** ]   **Cdk4**  p21WAF1 Cip1 complex formation.  Ref: 12796485 J Biol Chem< MedlineTA>J Biol Chem, 2003
- These inhibitors also effect strong G1 arrest in these cell lines and inhibit Rb phosphorylation at Ser780 consistent with inhibition of cyclin D1  [ **CCND1** ]   **CDK4** .  Ref: 12824014 Bioorg Med Chem Lett, 2003
- In the lactacystin treated OLGcs, there was a dose dependent decrease in the number of cells incorporating bromodeoxyuridine and in the activity of the complexes cyclin D  [ **CCND1** ]   **cdk4**  and cyclin E cdk2.  Ref: 12805303 J Neurosci, 2003
- In contrast, proliferative ZD Wt forestomach showed strong expression of Bcl  [ **CCND1** ]  2, an antiapoptotic protein, and overexpression of cyclin D1  [ **CCND1** ]   **Cdk4** .  Ref: 12873989 Cancer Res, 2003
- The high rate of cell proliferation was accompanied by overexpression of cell cycle progression and tumorigenesis biomarkers, including proliferating cell nuclear antigen, cyclin D1  [ **CCND1** ] , cyclin dependent kinase 4  [ **CDK4** ] , p53, cytokeratin 14, epidermal growth factor receptor, and by a reduced rate of apoptosis.  Ref: 12874033 Cancer ResCancer Res, 2003
- Quantitative immunochemical evaluation of the expression of certain proteins controlling the cell cycle showed an increase in p27kip1, cyclin D  [ **CCND1** ] , and  **cdk4**  positive cells, with a decrease in cyclin E and cdk2 positive cells.  Ref: 12805303 J Neurosci, 2003
- EGF administration after PH also significantly affected the activity of the cyclin D1  [ **CCND1** ]   **cdk4**  and cyclin E cdk2 complexes, mainly by changing their time progression it accelerated the increase in activity to 18h and caused a subsequent drop in activity after 34h.  Ref: 12876382 Cell Physiol Biochem, 2003
- Overexpression of cyclin D1  [ **CCND1** ] , the regulatory subunit of cyclin dependent kinases  **cdk4**  and cdk6 involved in cell cycle control, has often been found in breast cancer and other types of human cancer.  Ref: 12834607 J Comp Pathol, 2003
- Western blotting analysis revealed that the protein levels of cyclin A, cyclin E, and cyclin dependent kinase CDK 2 but not cyclin D1  [ **CCND1** ]  and  **CDK4**  decreased after progesterone treatment, but those of CDK inhibitory proteins, p21 and p27, increased.  Ref: 12810531 Endocrinology< MedlineTA>Endocrinology, 2003
- Novel, potent and selective cyclin D1  [ **CCND1** ]   **CDK4**  inhibitors indolo a pyrrolo c carbazoles.  Ref: 12824014 Bioorg Med Chem Lett, 2003
- Genetic characterization was performed using cytogenetics, fluorescence in situ hybridization, flow cytometry, and molecular techniques for analysis of the genes implicated in cell cycle control, amplification of MDM2,  **CDK4** , and Cyclin D1  [ **CCND1** ] , and mutations in the p53 gene.  Ref: 12808123 Lab Invest, 2003
- In our study, we screened HCCs resulting from HCV infection 51 cases, HBV infection 26 cases or excess alcohol intake 23 cases for alterations in genes involved in the RB1 pathway p16 INK4a, p15 INK4b, RB1,  **CDK4**  and cyclin D1  [ **CCND1** ] , the p53 pathway p53, p14 ARF and MDM2 and the Wnt pathway beta catenin, APC.  Ref: 12845670 Int J Cancer, 2003
